# Supplementary material for: Impact of pathogen genetics on clinical phenotypes in a population of Talaromyces marneffei from Vietnam
Source: Genetics. 2023 May 25;224(4):iyad100. doi: 10.1093/genetics/iyad100 (PMC10411598; doi:10.1093/genetics/iyad100)
Supplement: iyad100_Supplementary_Data [file iyad100_supplementary_data.zip › Supplemental_Figure_Legends_GENETICS-2023-306172.docx]

## **Supplemental Figure Legends**

**Figure S1. Recombination rates per chromosome.**

Recombination rates (p/bp) as calculated by Ldhelmet, for a subset of 50 isolates. For chromosomes 1-8, average p/bp values are: 0.00098, 0.00099, 0.00099, 0.00099, 0.00100, 0.00100, 0.00100, and 0.00101 respectively.

**Figure S2. Population structure and geographical clades.**

A) Population structure as determined by splitstree identifies two distinct clades, grouped by geography. B) Maximum likelihood phylogeny of patient isolates, with minimum inhibitory concentrations of itraconazole (ITR, blue) and amphotericin B (AMB, orange), visualized around the outer perimeter of the phylogeny.

**Figure S3. Linkage disequilibrium decay.**

Linkage disequilibrium decay over 250 kb for northern (orange) and southern (blue) clades, as well as all isolates combined (grey). LD50 is indicated by the dashed line.

**Figure S4. Initial fungal burden and clearance rate by clade.**

A) Rate of clearance (Slope) by the infecting isolate clade. Displayed as −1(gradient). B) Blood Log_10_ CFU/mL (fungal burden) by infecting isolate clade.

**Figure S5. Minimum inhibitory concentrations of itraconazole and amphotericin B.**

A) Histogram displaying the minimum inhibitory concentrations of itraconazole (ITR). B) Histogram displaying the minimum inhibitory concentrations of amphotericin B (AMB).
