# Supplementary material for: Impact of pathogen genetics on clinical phenotypes in a population of Talaromyces marneffei from Vietnam
Source: Genetics. 2023 May 25;224(4):iyad100. doi: 10.1093/genetics/iyad100 (PMC10411598; doi:10.1093/genetics/iyad100)
Supplement: iyad100_Supplementary_Data [file iyad100_supplementary_data.zip › Table_S1_GENETICS-2023-306172.docx]

#### Table S1. Functional enrichment of regions under selection across clades.

| Category | Test | GO Term | Adjusted p-value |
| --- | --- | --- | --- |
| Sub-telomeric | CLR | GO:0015980 | 0.04 |
| Sub-telomeric | CLR | GO:0042357 | 0.04 |
| Non sub-telomeric | CLR | GO:0015980 | 0.006 |
| Non sub-telomeric | CLR | GO:0045333 | 0.006 |
| Non sub-telomeric | CLR | GO:0015711 | 0.02 |
| Non sub-telomeric | CLR | GO:0006811 | 0.03 |
| Non sub-telomeric | CLR | GO:0010468 | 0.03 |
| Non sub-telomeric | Dxy | GO:0009061 | 0.05 |
