# Supplementary figures and images for: Impact of pathogen genetics on clinical phenotypes in a population of Talaromyces marneffei from Vietnam
Source: Genetics. 2023 May 25;224(4):iyad100. doi: 10.1093/genetics/iyad100 (PMC10411598; doi:10.1093/genetics/iyad100)

A

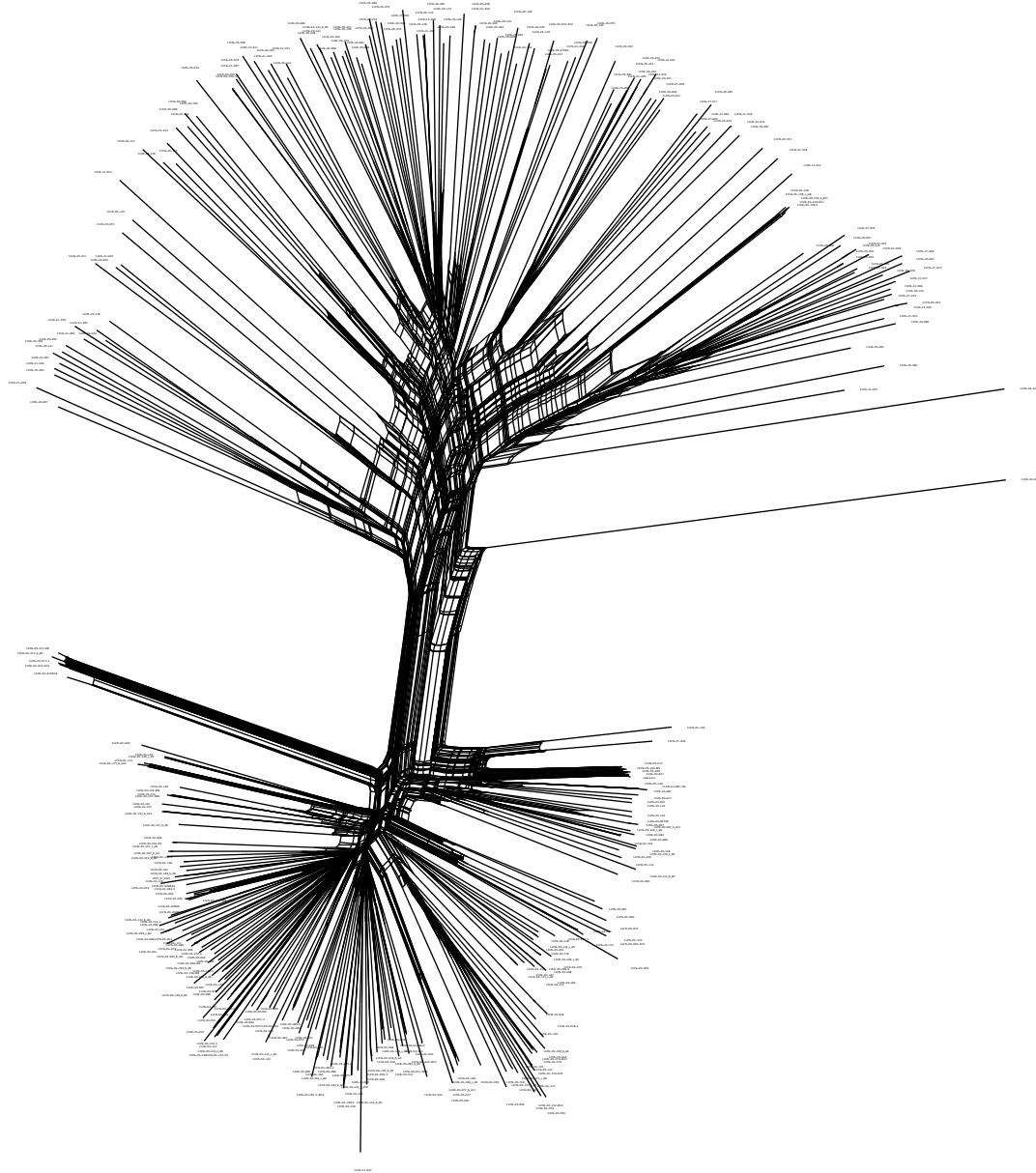

B

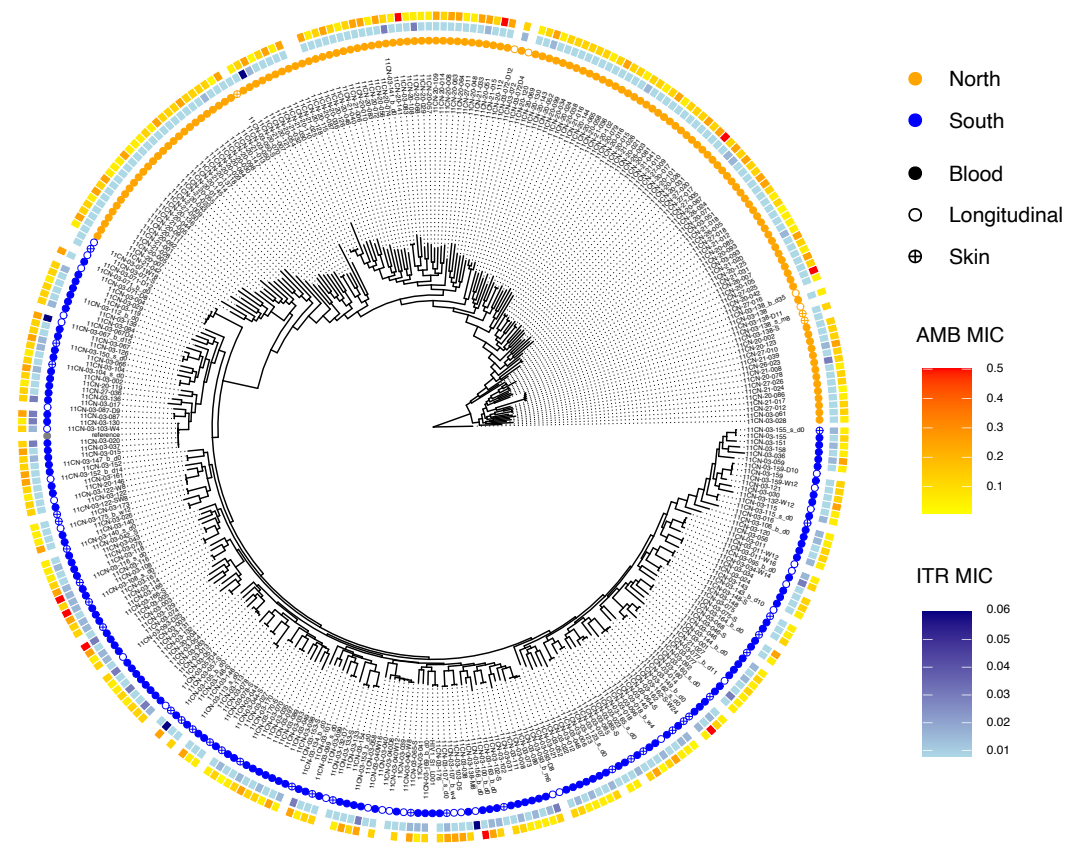

Supplement: iyad100_Supplementary_Data [file iyad100_supplementary_data.zip › Figure_S2_GENETICS-2023-306172.pdf]

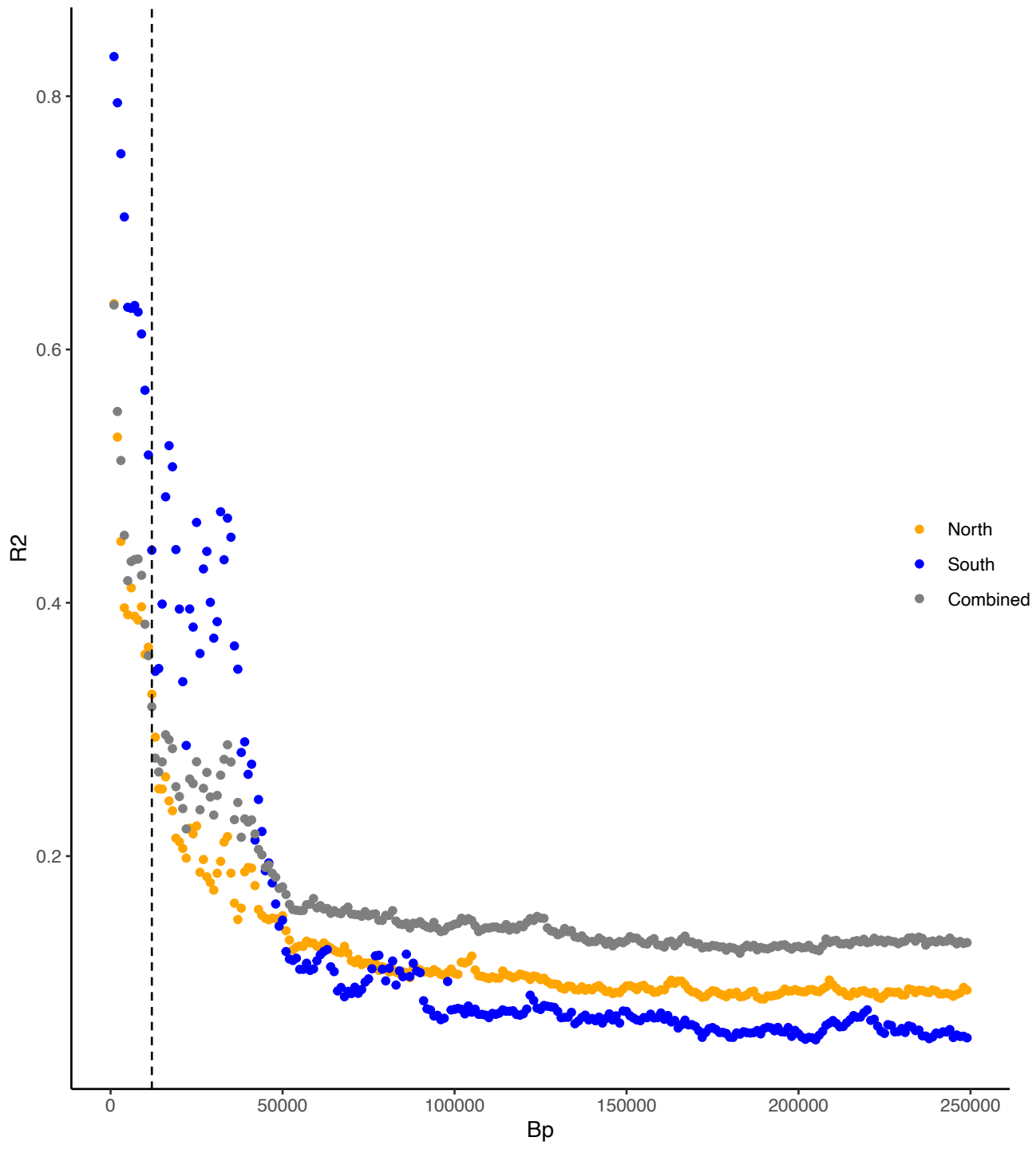

Supplement: iyad100_Supplementary_Data [file iyad100_supplementary_data.zip › Figure_S3_GENETICS-2023-306172.pdf]

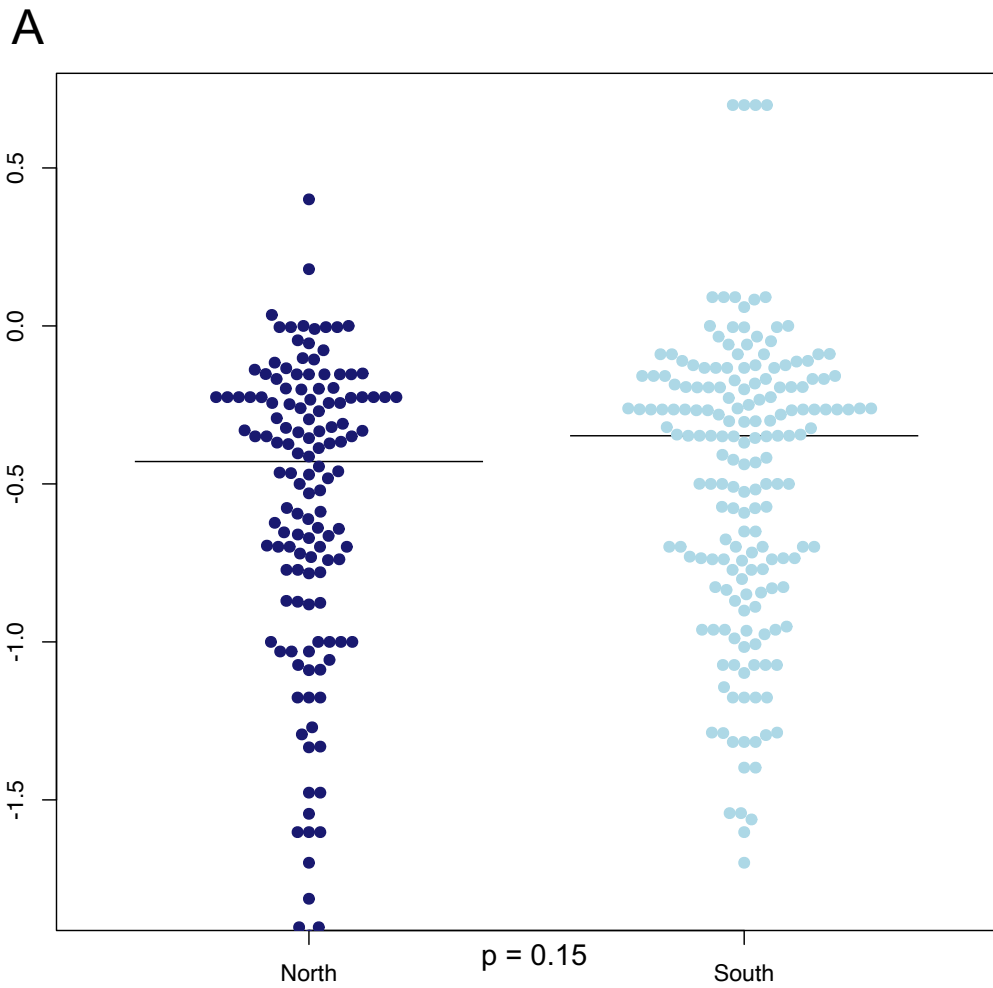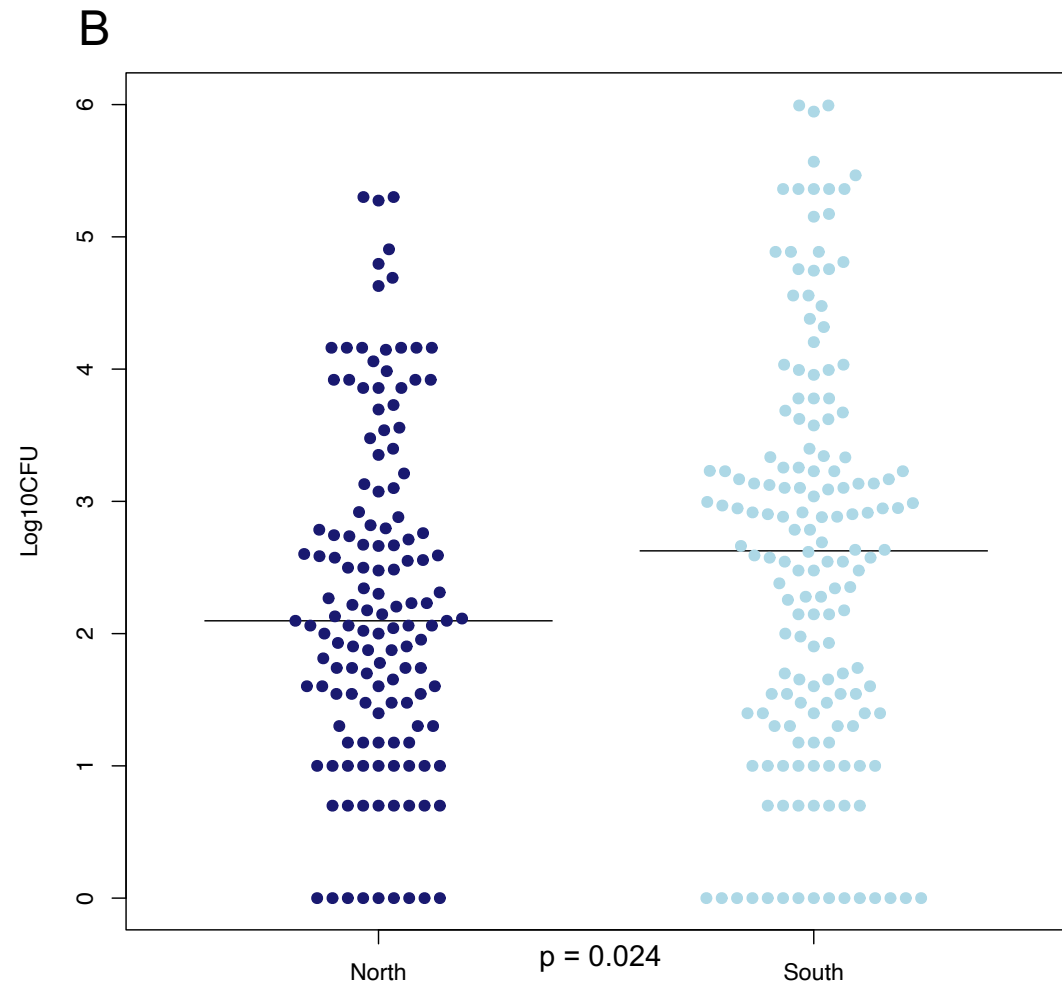

Supplement: iyad100_Supplementary_Data [file iyad100_supplementary_data.zip › Figure_S4_GENETICS-2023-306172.pdf]

**A**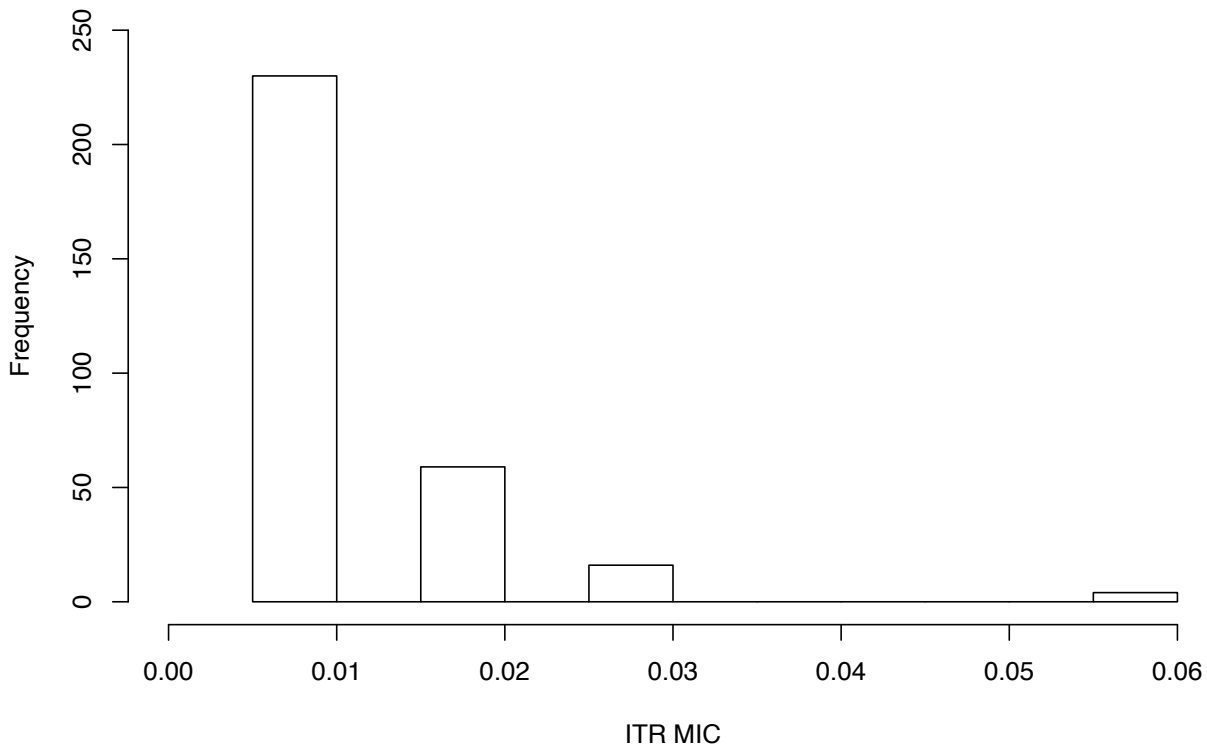**B**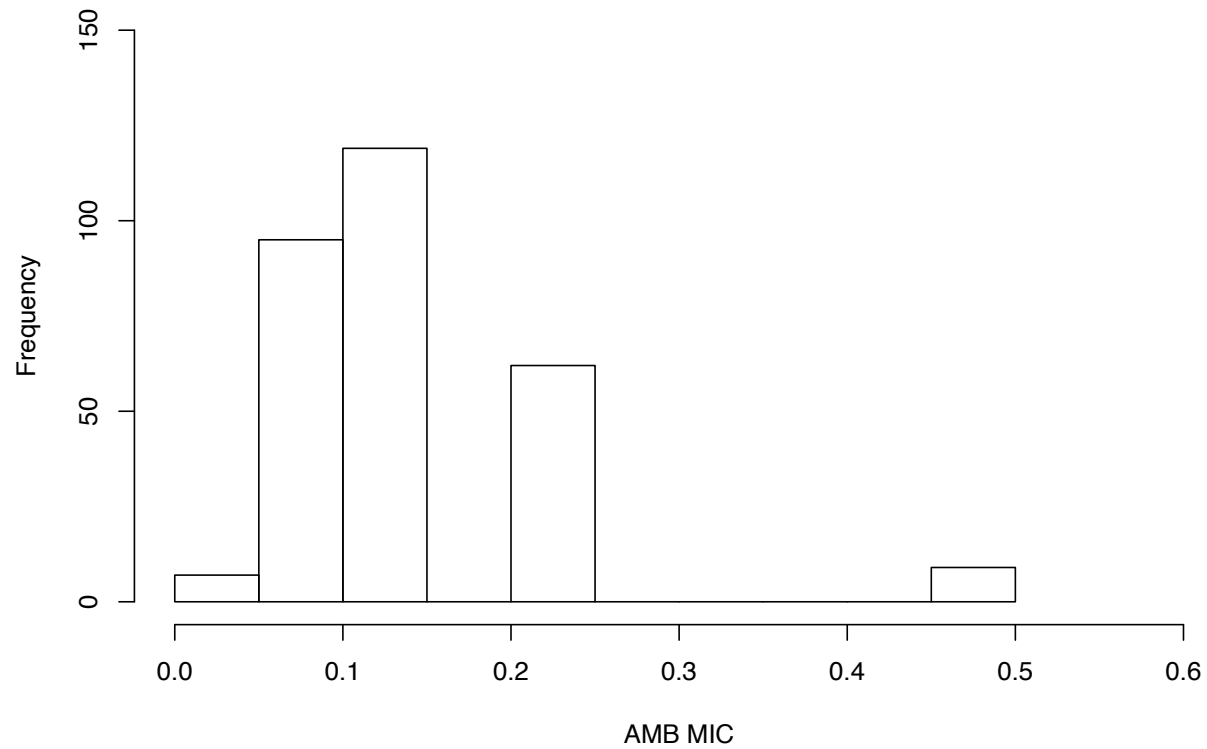

Supplement: iyad100_Supplementary_Data [file iyad100_supplementary_data.zip › Figure_S5_GENETICS-2023-306172.pdf]
