# Supplementary material for: Impact of pathogen genetics on clinical phenotypes in a population of Talaromyces marneffei from Vietnam
Source: Genetics. 2023 May 25;224(4):iyad100. doi: 10.1093/genetics/iyad100 (PMC10411598; doi:10.1093/genetics/iyad100)
Supplement: iyad100_Supplementary_Data [file Figure_S1_GENETICS-2023-306172.pdf › Figure_S1_GENETICS-2023-306172.pdf]

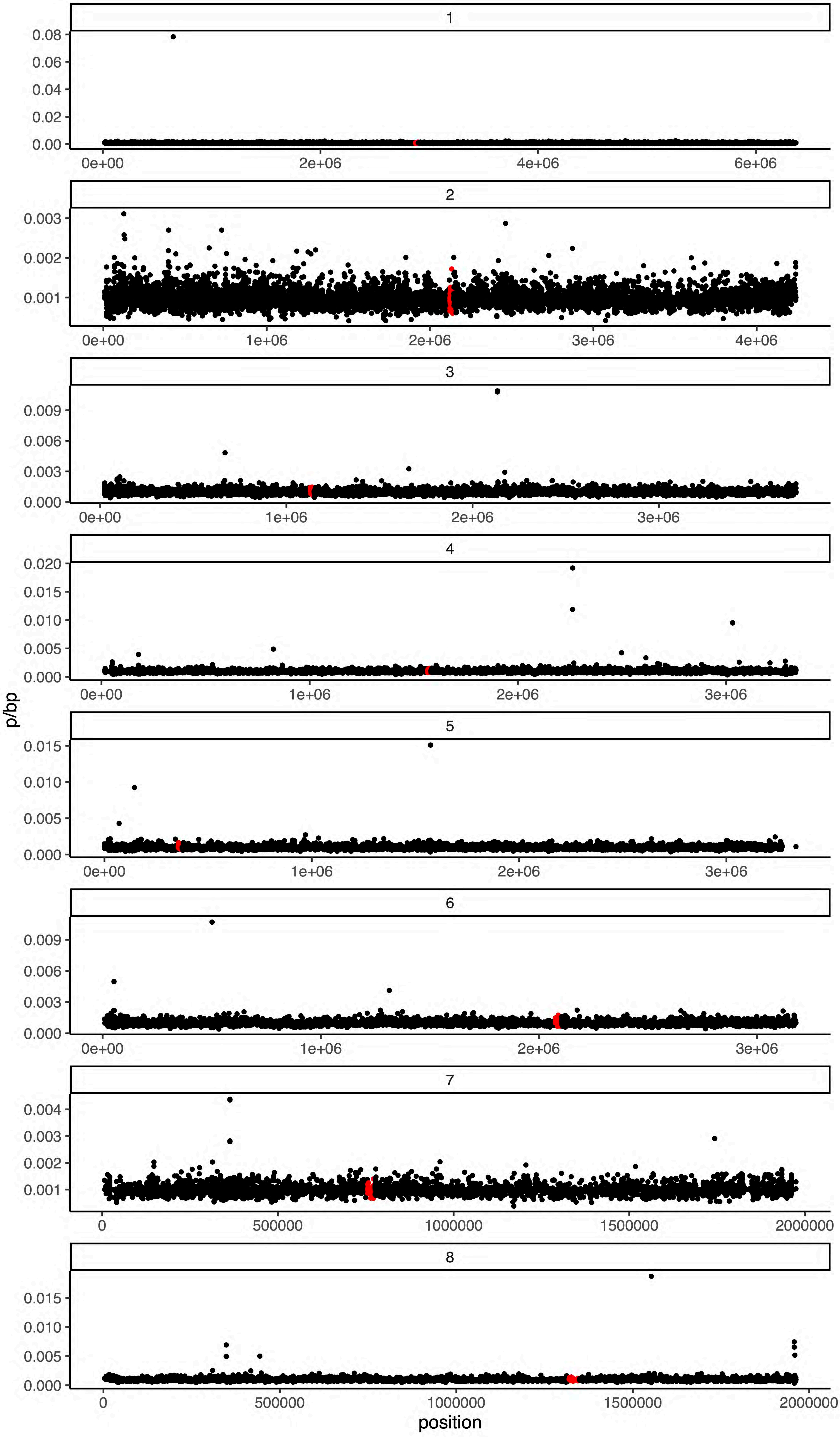

1
0.08
0.06
0.04
0.02
0.00
0e+00
2e+06
4e+06
6e+06
2
0.003
0.002
0.001
1e+06
3e+06
4e+06
3
0.009
0.006
0.003
0.000
4
0.020
0.015
0.010
0.005
5
6
7
0.004
0
500000
1000000
1500000
2000000
8
p/bp
position
